# Building the hospital event-based surveillance system in Viet Nam: a qualitative study to identify potential facilitators and barriers for event reporting

**DOI:** 10.5365/wpsar.2019.10.1.009

**Published:** 2020-09-30

**Authors:** Hien Do, Hien T Ho, Phu D Tran, Dang B Nguyen, Satoko Otsu, Cindy Chiu de Vázquez, Tan Q Dang, Quang D Tran, Van Anh Pham, Nanako Mikami, Masaya Kato

**Affiliations:** a World Health Organization Viet Nam Country Office, Viet Nam.; b Ha Noi University of Public Health, Viet Nam.; c General Department of Preventive Medicine, Ministry of Health, Viet Nam.; d Tohoku University Graduate School of Medicine, Japan.; e World Health Organization Regional Office for the Western Pacific, Philippines.

## Abstract

**Introduction:**

Hospitals are a key source of information for the early identification of emerging disease outbreaks and acute public health events for risk assessment, decision-making and public health response. The objective of this study was to identify potential facilitators and barriers for event reporting from the curative sector to the preventive medicine sector in Viet Nam.

**Methods:**

In 2016, we conducted 18 semi-structured, in-depth interviews, as well as nine focus group discussions, with representatives from the curative and preventive medicine sectors in four provinces. We transcribed the interviews and focus group discussions and used thematic analysis to identify the factors that appeared to affect public health event reporting.

**Results:**

We identified five major themes. First, the lack of a legal framework to guide reporting meant hospital staff relied on internal procedures that varied from hospital to hospital, which sometimes delayed reporting. Second, participants stated the importance of an enabling environment, such as leadership support and having focal points for reporting, to facilitate reporting. Third, participants described the potential benefits of reporting, such as support provided during outbreaks and information received about local outbreaks. Fourth, some challenges prohibited timely reporting such as not perceiving reporting to be the task of the curative sector and hesitancy to report without laboratory confirmation. Finally, limited resources and specialist capacities in remote areas hindered timely detection and reporting of unusual events.

**Discussion:**

This study identified potential opportunities to promote the detection and reporting of unusual events from health-care workers to the public health sector, and thus to improve the overall health security system in Viet Nam.The influenza virus is a respiratory pathogen that is transmitted through respiratory droplets.**^1^** During seasonal influenza epidemics, high attack rates cause a significant public health burden.**^2^** The infection is usually self-limited in young adults but can lead to severe infections in people in high-risk groups, including elderly people (> 65 years old), pregnant women, children aged 6–59 months and adults with chronic illnesses.**^3^**

Under the International Health Regulations, or IHR (2005), all Member States must develop core capacities to detect, assess, report and respond to acute public health events and emergencies. (*1*) For countries in the World Health Organization (WHO) South-East Asia and Western Pacific regions, the *Asia Pacific Strategy for Emerging Diseases and Public Health Emergencies* (APSED III) has served as the regional framework for action to guide Member States to advance the implementation of the IHR (2005) for health security. (*2*)

APSED III proposes incorporating health-care workers in the surveillance system as a priority for the early detection of public health threats. Lessons learnt from previous public health emergencies have highlighted the potential benefits of engaging health-care workers in the event-based surveillance (EBS) system for the rapid and timely detection of emerging diseases and public health emergencies. (*3*-*6*) APSED III further emphasizes using multiple sources of information, including event reporting from health-care facilities and laboratories during risk assessment to better inform decision-making. (*2*) In Viet Nam, the initial EBS system relied on media monitoring, and there was no systematic approach to promote timely reporting of public health events from health-care workers. (*7*) In view of this, there have been plans to expand the EBS system in Viet Nam.

Viet Nam has a well established notifiable disease surveillance and reporting system that is known, accepted and implemented by all levels of the health-care system – national, regional, provincial, district and commune levels. The reporting role relies on the curative (medicine) sector, which includes hospitals and other health-care facilities (both public and private), to report directly through an electronic reporting system and in coordination with the preventive medicine sector in their respective level – General Department of Preventive Medicine (GDPM) at the national level, Pasteur Institute or Institute of Hygiene and Epidemiology at the regional level, Provincial Preventive Medicine Centre (PPMC) or Provincial Centre of Disease Control (PCDC) at the provincial level, District Health/Preventive Medicine Centre (DPMC) at the district level, and Commune Health Station (CHS) at the commune level. While the curative sector is in charge of reporting disease and events, the preventive medicine sector is responsible for verification, investigation and response in coordination with the curative sector and other relevant stakeholders.

In this study, our overall goal was to gain insights into the current situation of event reporting from the curative sector and response from the preventive medicine sector, to inform broader system strengthening and to further engage health-care workers in the surveillance of public health threats. More specifically, we aimed to identify potential facilitators and barriers for signal detection, timely reporting and rapid response in the event of a public health emergency, which we hope to eventually use as the foundation to design a hospital EBS (HEBS) system in Viet Nam.

## Methods

### Study design

**Figure 1:**
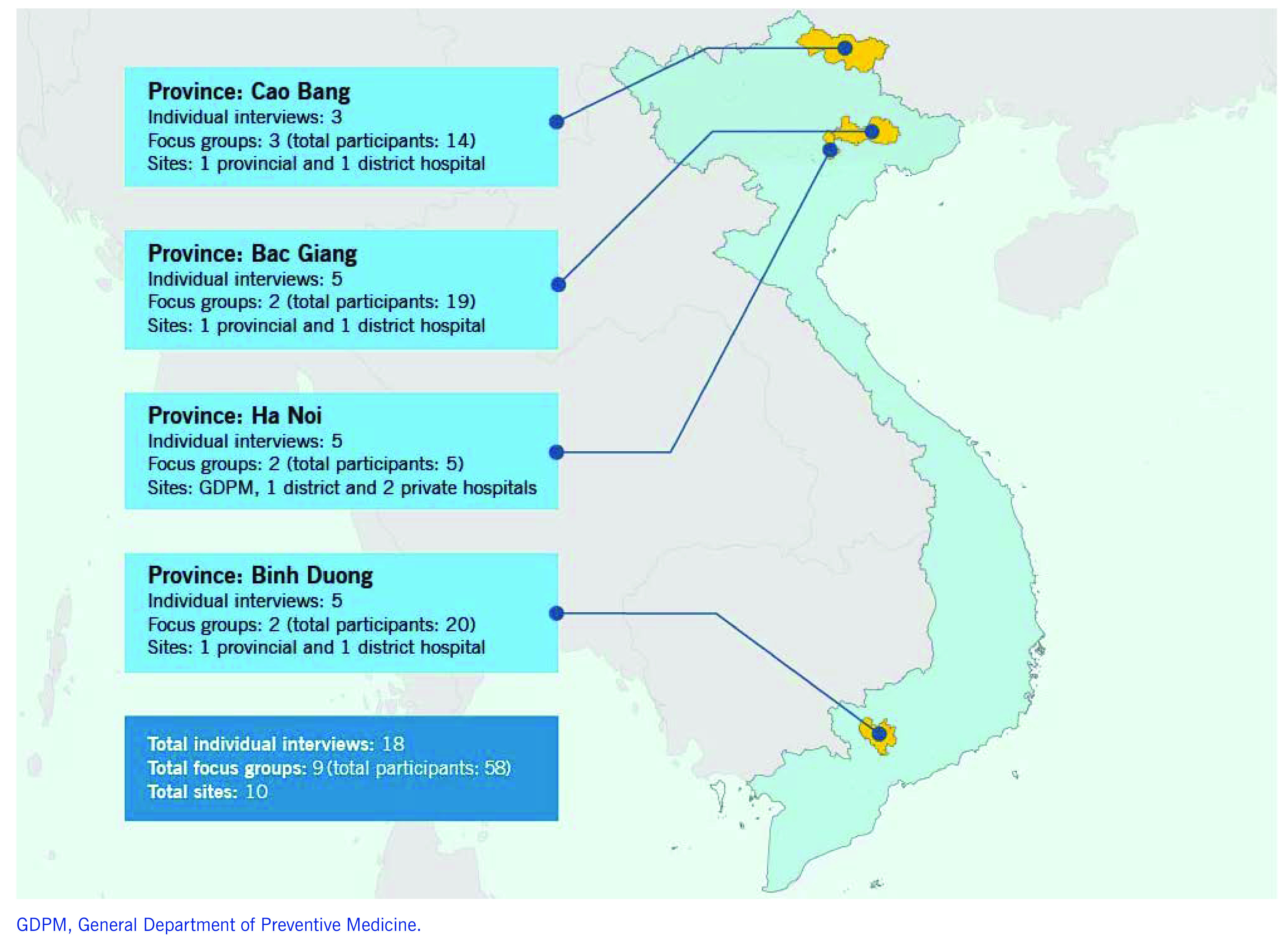
**Participating sites in the qualitative study to identify facilitators and barriers for event reporting from hospitals to the public health system, Viet Nam, 2016**

From July to December 2016, we conducted semi-structured individual interviews and focus group discussions with representatives from the curative and preventive medicine sectors to explore and understand the reporting of “unusual events” from the curative to the preventive medicine sector. We also reviewed documents and archival records as supplemental data. We employed a purposeful sampling strategy for the effective use of resources to allow data extraction from “information-rich cases” to yield “insights and in-depth understanding rather than empirical generalizations” as described by Patton in 2002. (*8*) We carried out this study in four provinces: Ha Noi (capital of Viet Nam), Bac Giang (northern Viet Nam), Cao Bang (mountainous, remote area) and Binh Duong (southern Viet Nam) (**Fig. 1**). One district was purposively selected for each province to conduct the study based on convenience, their level of cooperation, having had a recent disease outbreak or has the potential to have disease outbreaks.

### Participant characteristics

We conducted a total of 18 semi-structured, in-depth individual interviews and nine focus group discussions (with a total of 58 participants) (**Fig. 1**). Participants recruited in this study included hospital ward and laboratory staff who may detect unusual events for reporting; hospital leadership team members and planning department staff who are also the key decision-makers for determining the reporting process; and leaders and staff receiving reports at the preventive medicine centres (PMCs). We recruited participants from the GDPM (central governmental body in Ha Noi that oversees all PMCs), one DPMC, three provincial hospitals, four district hospitals and two private hospitals.

[**insert** Figure 1]

### Data collection and analysis

Informed consent was obtained before conducting the interviews and focus group discussions. Three different semi-structured interview guides were developed and used to interview medical doctors, laboratory staff and hospital leadership teams. The focus group guide was developed and used to guide the discussion for provincial/district preventive medicine staff and hospital staff. Topics covered included the current reporting practice of unusual events, awareness, attitudes, potential barriers and solutions, and lessons learnt. Specific hypothetical scenarios were also used to identify possible actions that health-care workers may take upon detection of an unusual event. In addition, we also reviewed training records, logbooks and reporting forms to supplement interview data. Interviews and focus groups were led by experienced qualitative researchers, conducted in Vietnamese, and lasted approximately 60–90 minutes each; digital recordings of the sessions were transcribed verbatim for thematic analysis, which was performed in the NVIVO 8.0 software (QSR International, Melbourne, Australia). We conducted the data analysis simultaneously with data collection and data interpretation, which was iterative throughout the research process. We first used open coding to inductively classify data into initial categories or themes, which was then followed by axial coding to examine the data for regularities and variations within and between themes. (*9*) The research team also met several times to discuss the key themes for verification and deepening the analysis of the results.

### Ethical approval for this study

This study has been reviewed and approved by the Institutional Ethical Review Board of Ha Noi University of Public Health in 2016.

## Results

Five main themes emerged during the focus group discussions and in-depth interviews (**Table 1**).

**Table 1:** Summary of key findings – the current situation for reporting “unusual events” from hospitals,  Viet Nam, 2016

Key findings				
**Legal framework and standard operating procedures may play an important role in guiding reporting and response.**	**An enabling environment is necessary for timely reporting and response.**	**Potential benefits exist for the curative sector to work with the preventive medicine sector.**	**Health-care providers face multiple challenges to timely reporting.**	**Extra challenges exist for signal detection and reporting from remote areas and industrial zones.**

SPECIFIC FINDINGS		
Signal detection	Timely reporting	Rapid response
**Challenges in detecting clusters despite awareness of “unusual events”** **Although hospital and laboratory staff were sufficiently aware of what constitutes an “unusual event,” they were not aware of other similar unusual cases in other departments or other hospitals; therefore, they could not recognize clusters within the hospital.** **An electronic reporting system may be one way to facilitate detection of clusters of similar cases through easy data sharing within the hospital.** **Some doctors reported that the provision of information on disease trends in the locality might also help them be more aware.** **Unfamiliarity with rare infectious diseases can result in missed signals.** **Some medical staff were not familiar with rare infectious diseases; therefore, they failed to detect signals or facilitate timely referral due to misdiagnosis.** **“Unusual events” were not considered signals for reporting until confirmatory diagnosis.** **There was a misconception of the need to have a confirmatory diagnosis before reporting. Some doctors reported fear of being judged if the unusual event reported turned out to be not unusual.** **With most provincial and district laboratories having insufficient capacity to perform the necessary diagnostic tests, doctors were hesitant to report “unusual events.”** **Delayed laboratory results also decreased the incentive to send samples for confirmation to the provincial preventive medicine laboratories.** **Additional challenges to detect signals in rural areas** **The situation was exacerbated in rural areas because of limited access to hospitals, fewer doctors trained in infectious diseases, limited laboratory capacity and cultural differences.**	**Supportive leadership and designated focal points were critical for timely reporting.** **Although timely reporting was challenging in hospitals with a hierarchical structure, it was facilitated in other hospitals with supportive leadership. Overall, reporting systems worked best in hospitals with a designated focal person and backup focal persons assigned for reporting.** **Minimal ownership of reporting tasks among hospital staff** **Following the introduction of Circular 54, which transferred the reporting task from the preventive medicine sector to the curative sector, some hospital staff have yet to fully accept this change.** **Medical doctors were reluctant to report “unusual events” as they were not familiar with this activity and found it complicated and time-consuming. In addition, the reporting procedure was unclear in some hospitals.** **Unidirectional reporting reduced incentives to report.** **Some hospital staff felt reporting to the preventive medicine sector was mostly a one-way relationship. A lack of feedback from the preventive medicine sector can decrease their incentives to report.** **Timely reporting was seen in hospitals with close links to the preventive medicine sector and regular two-way communication.** **Rapid response from the preventive medicine sector after receiving a report also enhanced the hospital staff’s desire to report.** **Extra difficulties for reporting from rural areas and industrial zones** **Some areas, especially industrial zones, may not report because of the lack of or unclear reporting requirements/enforcement, and fear of economic ramifications.**	**Designated 24/7 focal points in the preventive medicine sector can facilitate rapid response.** **Focal points assigned in the preventive medicine sector to receive reports from hospitals can facilitate rapid public health responses.** **It is ideal to appoint backup focal points when focal points are not available so hospitals can report 24/7.** **Rapid response from the preventive medicine sector can build trust and a collaborative relationship between the two sectors.** **Rapid response is not possible unless the preventive medicine sector is informed of possible public health events in a timely manner.** **However, once reports are received, rapid response from the preventive medicine sector can also help hospital staff to see the value in timely reporting, thus improving their attitude and cooperation towards reporting.**

### Theme 1 – Legal framework and standard operating procedures may play an important role in guiding reporting and response

Hospital staff reported the lack of a legal framework and standard operating procedures (SOPs) as challenges that hinder event reporting. At the time of the study, no legal framework or national guidelines on EBS in Viet Nam existed. Although some institutions have their own internal reporting procedure, many do not. Some participants expressed their desire to have a more formalized system in place, as one hospital staff stated:

“At present, we haven’t got an official system to enable hospital departments to easily share information with provincial preventive medicine centres. So I think we should have a system in the future. I support this idea.”

Furthermore, no legal process is in place to mandate reporting. In non-residential industrial zones where there are only enterprises, manufacturers and companies producing industrial products and services, some companies reportedly tried to hide disease outbreaks among their employees due to the potential economic impact. Respondents reported that companies do not see it as their responsibility to report to the health authority, as explained by one PMC staff:

“The Department of Health at the provincial level needs to inform all companies to report infectious diseases to them. They need to inform our disease control department. They might hide an unusual outbreak [or] ignore it because they are afraid the media will announce the disease. We only [know] after they bring their family member to the hospital for treatment.”

### Theme 2 – An enabling environment is necessary for timely reporting and response

Hospital staff described several factors in their work environment that promote timely reporting.

**Supportive leadership.** In hospitals with supportive leadership who believed in the value of reporting, timely reporting was not an issue. However, in hospitals with strict hierarchal reporting structures, staff may be punished, as one doctor explained:

“In case I report to the planning department without informing our head of department, the hospital director might ask, ‘Oh, what happened, does the head of the department know?’, and if he didn’t know, I am in trouble.”

**Availability of internal procedures to guide reporting.** Limited guidance regarding the reporting process can create confusion among hospital staff. In hospitals with internal procedures, reporting was better implemented, as one doctor described:

“If I find an unusual case, first, we will discuss within our department. Then, I will report to the head of our department to confirm the case is unusual and requires further reporting. If it is, we will report to the leader who is in charge of that shift, or report to the planning department so that they can inform systematically.”

**Good personal relationships between hospitals and PMCs.** In provinces where there were good personal relationships between hospitals and PMCs, we saw enhanced crosstalk and event reporting. As one PMC staff explained:

“The hospital often calls me if there is something unusual, no matter if it’s during or after working hours. They call me often; it is not under any system yet. … If something happens, we have to get a sample, so we send a person there straight away to get a sample. Then we will investigate the situation, perform tests quickly, and help them as soon as possible. After investigating at the hospital, we have to investigate the community as well.”

**Assigned focal points at hospitals and PMCs to facilitate rapid information exchange.** One key factor of success for prompt notification of unusual events has been assigning focal points at hospitals and PMCs. As one hospital staff mentioned:

“One person at PMC is assigned to take care of each hospital or area. This is one favourable factor. They have an administrative landline and mobile to contact when they need. It’s important to have the responsible person to inform. We can report to the leader later. It’s quicker to inform the preventive medicine sector.”

### Theme 3 – Potential benefits exist for the curative sector to work with the preventive medicine sector

Hospital staff reported several potential benefits or factors that could prompt them or encourage them to report unusual events and work with the preventive medicine sector.

**Outbreak response and containment.** One incentive to work with the preventive medicine sector is the support provided by PMCs during outbreaks. By informing PMCs of suspected outbreaks, hospitals are more likely to experience a timely response and outbreak containment, reducing the burden on hospitals. As one doctor explained:

“It is necessary to have prompt and quick actions to facilitate timely medical consultation and exams for more effective treatments. This would help the occurrence of outbreaks that we could prevent. We can then have a prompt response when an outbreak occurs.”

**Knowledge of the local outbreak situation can increase doctors’ awareness and improve diagnosis, treatment and care.** Knowing the epidemiological situation may help doctors in their clinical practice; however, reporting is often unidirectional, with no or limited feedback received after reporting. The same doctor summed up the reporting direction with PMC in one sentence:

“We only report to them; we do not receive feedback from them.”

**Laboratory confirmation by the preventive medicine network.** Some doctors noted that confirmatory laboratory results help with diagnosis and treatment, and provide external feedback to hone clinical skills. Since hospital laboratory capacities are limited, clinicians benefit from PMC-facilitated laboratory testing through their laboratory network. One doctor explained:

“We are clinical doctors; we want to have experience in diagnosis and treatment. We want to know how accurate our diagnoses are.”

However, laboratory testing in the preventive medicine sector is mainly for surveillance purposes. For diagnostic testing, long delays in receiving results preclude their use in patient diagnosis or treatment. Another doctor said:

“The preventive medicine centre delays the release of test results. I don’t know the reasons why, but they provide results so late that the patients have already been discharged. As a treating doctor, it’s difficult to treat a patient without having a confirmatory diagnosis.”

### Theme 4 – Health-care providers face multiple challenges to timely reporting

Hospital staff reported several challenges that prohibited timely event reporting from the curative sector.

**Reporting is not perceived to be the responsibility of hospital staff.** Many doctors believe their focus should be on treatment and do not perceive reporting to be the task of the curative sector. Some doctors also think they are too busy to do reporting and are not familiar with reporting tasks. One doctor summed it up:

“It’s more appropriate to ask the preventive medicine sector to do reports.”

**Hospital staff do not see the value of reporting.** Many doctors do not see the value or importance of reporting and how it can benefit them. Therefore, they do not prioritize the task of reporting. One doctor explained:

“We have to do all different things; we don’t report straight away if we have too many things.”

**No guidance or formal mechanisms in place.** In the absence of national guidance and formal mechanisms, some hospitals have opted to have their own internal reporting procedure. This may require first getting approval by the Department of Planning before reporting to the preventive sector, which can delay timely reporting of an unusual event. As one hospital staff described:

“We collect cases every day and report to the hospital leaders before 7 PM. At the department level, we need to make a weekly report to send to the planning department; the planning department is in charge of sending it to the provincial department of health and the preventive medicine centre. They also check if the report is correct.”

**Hesitancy to report unless laboratory confirmation is available.** Many doctors have a fear of being wrong or judged if a reported case turned out to be “not unusual.” Consequently, many doctors only want to report when laboratory confirmation is available. As one doctor explained:

“If later, after we’ve reported, the department of health finds out that the disease is just a normal or a common case, we are afraid that they will turn around and ask us why we could not diagnose an easy case.”

### Theme 5 – Extra challenges exist for signal detection and reporting from remote areas and industrial zones

Hospital and PMC staff reported additional difficulties in remote areas.

**Limited resources and experience.** In remote areas, some clinicians found it difficult to recognize uncommon diseases. There is also a lack of local laboratory facilities; thus, treatment decisions were based solely on clinical judgment. For example, a hospital staff described a case of a patient with Coxsackie virus infection in a remote area who was neither referred to the infectious diseases department nor reported.

“The patient was only 4 months old. The patient had respiratory distress, so it was very hard to categorize. The treatment department said that the patient should be in the neurology department; it doesn’t matter if he has an infectious disease or not. We still face difficulties in categorizing patients, so we did not report.”

Another hospital staff reported a cluster of children with pertussis that was misdiagnosed as leukaemia given their unfamiliarity with the condition.

“There were several kids with the same cough and tests. At that time, I didn’t know what pertussis was like. … We didn’t think of pertussis because we haven’t seen [cases] for a very long time, so it’s very sudden. ... I didn’t know what to do. I explained to their families that it could be leukaemia, so we sent them to the Provincial General Hospital. The hospital did the same thing and sent them to the National Hematology and Blood Transfusion Hospital in Ha Noi. The doctors in the hospital witnessed the cough after two days; they thought it could be pertussis and treated the patient for pertussis. After that we had more and more similar patients coming.”

**Differences in language and culture.** Other issues such as distance, language barriers and cultural barriers can also hinder early detection and timely reporting. One PMC staff mentioned the need to use law enforcement at times to “force” people who resisted medical care due to cultural reasons to go seek health care.

“We have to use law enforcement and follow the law of infectious diseases. The community did special things for the people who died, so people cannot go inside the houses during the three days after death. No one was allowed to go in. They would say, ‘This is my child, not yours, [and] even if they die, I can just give birth again.’ It’s complicated. So for our people’s health, we have to be strong, determined and do law enforcement. Sometimes we even had to ask [the] police to help in forcing them to go to the hospital for treatment.”

## Discussion

We found that hospital and laboratory staff were generally aware of what constituted an “unusual event.” Our study further identified facilitators and barriers to timely reporting. Close relationships between hospitals and PMCs facilitated timely reporting and rapid public health response. In addition, instituting focal points at the hospitals and PMCs further clarified roles and responsibilities and facilitated the reporting process. Key issues that hindered early detection and timely reporting included clinicians not considering reporting as their role; uncertainty regarding the reporting process; a lengthy approval process for reporting in some hospitals; hesitancy to report before confirmatory diagnostic testing; and challenge in recognizing clusters within the hospital. The one-way reporting process with minimal feedback from the preventive medicine sector also discouraged reporting.

### Fostering a “win-win” relationship between health-care and public health systems

A common theme seen in this study and previous studies in other contexts was the need to strengthen the relationship between the curative and preventive medicine sectors to ensure two-way communication. (*3*) Although most studies investigating different ways to motivate reporting were for routine reporting through the indicator-based surveillance (IBS) system, we believe it is also applicable to reporting rare and unusual events through the EBS system.

To facilitate reporting from health-care workers to the public health system, health-care workers also need tangible benefits of working with the public health system. In other words, it is important to foster a “win-win” relationship between health care and public health systems. Some study participants believed that information on national and local outbreaks would help their ability to diagnose and treat their patients. Previous studies have recommended generating a feedback report and ensuring they reach reporters, so they see the value in reporting. (*10*-*12*) Other studies have shown that tailoring feedback to focus on the current outbreaks and other information of interest to medical staff can also encourage reporting. (*13*) We also believe this may be an important approach that promotes ownership.

Laboratory services at PMCs may also help physicians at health-care facilities with their clinical practice in the diagnosis of infectious diseases. We did note that depending on the province, some laboratory services in the preventive medicine sector were not able to fulfil the physicians’ expectations. We believe streamlining preventive medicine laboratory services could contribute to the strengthening of the working relationship of the health-care and public health systems, and thus, in turn, promote the early detection of outbreaks and public health events.

### Raising awareness on the value of reporting

Many medical professionals in our study did not perceive that reporting events was their responsibility. In addition, they were not fully aware of what, how and when to report, as we have seen in other countries. (*3*, *10*, *11*, *14*) Passive attitudes, lack of knowledge regarding reporting requirements and misconceptions regarding the value of reporting seen in our study have been previously observed. (*3*, *10*, *11*, *14*, *15*) Certain beliefs, knowledge and attitudes held by physicians, such as the ones we saw in our study, are associated with underreporting. (*15*) These findings point to the need to raise awareness of the value of reporting among medical staff.

For some medical professionals, it may be difficult to recognize the importance of a rare event. One possible strategy is to present scenarios and lessons learnt from past outbreaks such as the 2015 Middle East respiratory syndrome outbreak in the Republic of Korea, which dampened economic growth and impacted the reputation of some hospitals. Describing the role of medical professionals in these past outbreaks may help providers embrace their unique position as the guardians at the first line of defence. (*16*, *17*) A previous study has also suggested the use of financial incentives or a penalty system to encourage reporting. (*11*) Different approaches to motivate reporting among medical staff, specifically in Viet Nam, may need to be explored. With a longer vision in mind, a strong sense of ownership and expanded responsibility of their role as reporters may need to be cultivated during the training and sensitization process.

### Creating an enabling environment for reporting

Although raising awareness among medical staff may increase their motivation to report, individual motivation depends on an enabling environment that facilitates reporting. In this study, participants expressed a lack of knowledge of the reporting process. Study participants also revealed that the reporting process could be lengthy, given the layers of approval required at many hospitals. Hospitals that empower their staff to report immediately to the PMC are the minority. We believe that creating an enabling environment is critical for the success of an EBS system. An enabling environment includes clear guidelines that designate a responsible focal person, describe the roles and responsibilities and lines of reporting, and establish goals and expectations. In addition, staff should be provided with the means needed to report and have leadership support to ensure that the responsible person has protected time allocated for reporting activities. Finally, training of new staff and regular re-training of existing staff may be an important way to continually sensitize the medical staff. (*18*)

### Promoting a simple and flexible reporting process

Developing a process for event reporting that is appropriate for all 63 provinces in Viet Nam is challenging, given their differences in resources and workforce capacity. Therefore, keeping the system flexible, and having the ability to tailor the system to the capability of each province, may be one of the key factors for success. Previous studies have shown that the simplicity of the reporting system is one of the most important factors to encourage reporting from clinicians. (*10*, *11*, *13*, *14*)

### Limitations

The findings in this study represent only the views of the purposefully selected hospital and preventive medicine staff in four provinces in Viet Nam; therefore, the generalizability of the findings may be limited. In addition, this was an exploratory study carried out for public health practice, which we focused on obtaining in-depth insights and synthesizing the information from all sources into key themes that were actionable instead of a comparative analysis study. Therefore, we did not present on the differences between the provinces, or the responses from key informants who were in different roles or at various levels of the organization. For the same reasons, a certain level of flexibility was required in sample selection; depending on facility size and availability of staff, in rare occasions, focus group discussions also had fewer than five participants.

## Conclusions

In this study, we showed that an enabling environment is critical for timely event reporting. This encompasses multiple components such as having leadership support, a good relationship between the two sectors, clear guidance on the process of reporting, and focal points to streamline reporting. However, we believe the fundamental key to success for both IBS and EBS is cultivating a “win-win” relationship between the curative and preventive medicine sectors, where both sides can see the value and benefits of this synergistic collaboration. Moving forward, as outlined in **Table 2**, we believe there are priority actions that can be taken to strengthen this important relationship further and ultimately to improve the overall health security system in Viet Nam.

**Table 2:** Proposed key interventions to strengthen hospital event-based surveillance system in Viet Nam based on the key findings from the qualitative study conducted in 2016

Overall recommendations			
**Develop a legal framework, guidelines and SOPs.**	**Promote feedback from the preventive medicine sector and communication within the curative sector.**	**Streamline preventive medicine laboratory services to support signal detection and timely reporting.**	**Build technical capacity in signal detection, reporting and response through training and on-the-job coaching during monitoring visits.**

Proposed key interventions			
**Develop clear guidelines and SOPs for both hospital internal reporting and reporting to PMCs.** **Engage DOH in encouraging collaboration between the curative and preventive medicine sectors.** **Review and identify opportunities to improve existing guidelines and SOPs for the preventive medicine sector.** **Improve current feedback reporting template and procedure in the preventive medicine sector to promote two-way communication with the curative sector.** **Pilot-test guidelines and SOPs in selected provinces to inform the development and implementation of the national EBS guidelines in Viet Nam.**	**Strengthen and implement regular feedback reporting from the preventive medicine sector to the curative sector to demonstrate how reported data are used and to inform disease trends in the locality.** **Promote a close relationship and communication between the two sectors by assigning a dedicated focal person and backup focal persons at the hospital (responsible for reporting) and at the PMC (responsible for receiving and responding to reports).** **Encourage regular sharing of information on unusual cases in hospitals to identify clusters within the hospital in a timely manner, including during morning meetings, through local e-mail networks or by phone.**	**Streamline preventive medicine laboratory services, including defining their roles of referring specimens through their preventive medicine laboratory networks, and clarify their roles to health-care workers.** **Review laboratory result feedback systems and identify ways to increase turnaround time to incentivize medical practitioners to send specimens for diagnostic confirmation.** **Encourage a proactive follow-up of laboratory results and feedback.** **Identify ways for preventive medicine laboratories to provide regular updates to the hospitals regarding existing and new services available and feasible turnaround times.** **Review existing signals to be reported by the laboratory to increase the sensitivity of signal detection at the hospital laboratories.**	**Conduct training and refresher training for hospital and laboratory staff to sensitize them on the concepts of EBS, list of potential signals and reporting procedures.** **Include senior leadership in the training for hospital staff to encourage direct and timely reporting.** **Train surveillance staff in the preventive medicine sector on SOPs and epidemiological analysis to improve data analysis skills, and provide technical support as needed to PPMC to generate feedback reports.** **Implement periodic monitoring to provide on-the-job coaching to staff to increase their technical capacity in signal detection and reporting at the hospitals.** **Conduct additional monitoring visits and on-the-job coaching at hospitals and PMCs for rural areas and industrial zones.**

DOH, Department of Health; EBS, event-based surveillance system; PMC, preventive medicine centre; PPMC, Provincial Preventive Medicine Centre; SOP, standard operating procedure.

## References

[1] International Health Regulations (2005). 3rd ed. Geneva: World Health Organization; 2016.

[2] Asia Pacific Strategy for Emerging Diseases and Public Health Emergencies (APSED III). Advancing implementation of the International Health Regulations (2005). Manila: World Health Organization Regional Office for the Western Pacific; 2017.

[3] MacDonald E, Aavitsland P, Bitar D, Borgen K. Detection of events of public health importance under the international health regulations: a toolkit to improve reporting of unusual events by frontline healthcare workers. BMC Public Health. 2011 9 21;11(1):713. 10.1186/1471-2458-11-713 21936937 PMC3188493

[4] Xu BY, Low SG, Tan RT, Vasanwala FF. A case series of atypical presentation of Zika Virus infection in Singapore. BMC Infect Dis. 2016 17;16(1):681. doi:10.1186/s12879-016-2032-y pmid:27855636. 10.1186/s12879-016-2032-y PMC5112881 27855636

[5] Ho ZJM, Hapuarachchi HC, Barkham T, Chow A, Ng LC, Lee JMV, et al.; Singapore Zika Study Group. Outbreak of Zika virus infection in Singapore: an epidemiological, entomological, virological, and clinical analysis. Lancet Infect Dis. 2017 8;17(8):813–21. 10.1016/S1473-3099(17)30249-9 28527892

[6] Ohara H. Experience and review of SARS control in Vietnam and China. Trop Med Health. 2017;32(3):235–40. Available from https://www.jstage.jst.go.jp/article/tmh/32/3/32_3_235/_pdf 10.2149/tmh.32.235

[7] Decision: approving “event-based surveillance (EBS) procedures.” No. 134/QD-DP. Hanoi: General Department of Preventive Medicine, Viet Nam Ministry of Health; 2014.

[8] Patton MQ. Qualitative research and evaluation methods. 3rd ed. Thousand Oaks (CA): Sage Publications; 2002.

[9] Ezzy D. Qualitative analysis: practice and innovation. Crows Nest (NSW): Allen and Unwin; 2002.

[10] Konowitz PM, Petrossian GA, Rose DN. The underreporting of disease and physicians’ knowledge of reporting requirements. Public Health Rep. 1984 Jan-Feb;99(1):31–5. 6422492 PMC1424528

[11] Tan HF, Yeh CY, Chang HW, Chang CK, Tseng HF. Private doctors’ practices, knowledge, and attitude to reporting of communicable diseases: a national survey in Taiwan. BMC Infect Dis. 2009 1 29;9(1):11. 10.1186/1471-2334-9-11 19178741 PMC2642829

[12] Dagina R, Murhekar M, Rosewell A, Pavlin BI. Event-based surveillance in Papua New Guinea: strengthening an International Health Regulations (2005) core capacity. West Pac Surveill Response. 2013 7 30;4(3):19–25. 10.5365/wpsar.2013.4.2.001 24319609 PMC3854102

[13] Krause G, Ropers G, Stark K. Notifiable disease surveillance and practicing physicians. Emerg Infect Dis. 2005 3;11(3):442–5. 10.3201/eid1103.040361 15757561 PMC3298248

[14] Friedman SM, Sommersall LA, Gardam M, Arenovich T. Suboptimal reporting of notifiable diseases in Canadian emergency departments: a survey of emergency physician knowledge, practices, and perceived barriers. Can Commun Dis Rep. 2006 9 1;32(17):187–98. 16989044

[15] Figueiras A, Lado E, Fernández S, Hervada X. Influence of physicians’ attitudes on under-notifying infectious diseases: a longitudinal study. Public Health. 2004 10;118(7):521–6. 10.1016/j.puhe.2003.12.015 15351226

[16] Ki M. 2015 MERS outbreak in Korea: hospital-to-hospital transmission. Epidemiol Health. 2015 7 21;37:e2015033. 10.4178/epih/e2015033 26212508 PMC4533026

[17] Lee SI. Costly lessons from the 2015 Middle East respiratory syndrome coronavirus outbreak in Korea. J Prev Med Public Health. 2015 11;48(6):274–6. 10.3961/jpmph.15.064 26639740 PMC4676647

[18] Turnberg W, Daniell W, Duchin J. Notifiable infectious disease reporting awareness among physicians and registered nurses in primary care and emergency department settings. Am J Infect Control. 2010 6;38(5):410–2. 10.1016/j.ajic.2009.07.013 20031271

